# Precision N‐glycoproteomics reveals elevated LacdiNAc as a novel signature of intrahepatic cholangiocarcinoma

**DOI:** 10.1002/1878-0261.13147

**Published:** 2021-12-18

**Authors:** Jun Li, Ting Zhao, Jing Li, Jiechen Shen, Li Jia, Bojing Zhu, Liuyi Dang, Chen Ma, Didi Liu, Fan Mu, Liangshuo Hu, Shisheng Sun

**Affiliations:** ^1^ College of Life Science Northwest University Xi'an China; ^2^ Department of Hepatobiliary Surgery Institute of Advanced Surgical Technology and Engineering The First Affiliated Hospital of Xi'an Jiaotong University China

**Keywords:** glycopeptides, glycoproteins, glycoproteomics, hepatocellular carcinoma, intrahepatic cholangiocarcinoma, mass spectrometry

## Abstract

Primary liver cancer, mainly comprising hepatocellular carcinoma (HCC) and intrahepatic cholangiocarcinoma (ICC), remains a major global health problem. Although ICC is clinically different from HCC, their molecular differences are still largely unclear. In this study, precision N‐glycoproteomic analysis was performed on both ICC and HCC tumors as well as paracancer tissues to investigate their aberrant site‐specific N‐glycosylation. By using our newly developed glycoproteomic methods and novel algorithm, termed ‘StrucGP’, a total of 486 N‐glycan structures attached on 1235 glycosites were identified from 894 glycoproteins in ICC and HCC tumors. Notably, glycans with uncommon LacdiNAc (GalNAcβ1‐4GlcNAc) structures were distinguished from their isomeric glycans. In addition to several bi‐antennary and/or bisecting glycans that were commonly elevated in ICC and HCC, a number of LacdiNAc‐containing, tri‐antennary, and core‐fucosylated glycans were uniquely increased in ICC. More interestingly, almost all LacdiNAc‐containing N‐glycopeptides were enhanced in ICC tumor but not in HCC tumor, and this phenomenon was further confirmed by lectin histochemistry and the high expression of β1‐4 GalNAc transferases in ICC at both mRNA and protein expression levels. The novel N‐glycan alterations uniquely detected in ICC provide a valuable resource for future studies regarding to the discovery of ICC diagnostic biomarkers, therapeutic targets, and mechanism investigations.

## Graphical abstract

N‐glycans structures, N‐glycosites and N‐glycoproteins, were simultaneously identified and quantified in intrahepatic cholangiocarcinoma (ICC) and hepatocellular carcinoma tumors. The glycoproteomic analysis systematically revealed their commonly and differentially altered site‐specific glycans. Uncommon LacdiNAc structures were distinguished from other glycan isomers and their specific increase in ICC was discovered.

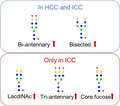

**Abbreviations**
ACN: acetonitrile
AFP: alpha fetoprotein
B4GALNT3: β1‐4‐acetylgalactosamine transferases 3
B4GALNT4: β1‐4‐acetylgalactosamine transferases 4
FFPE: formalin‐fixed paraffin‐embedded
GO: Gene Ontology
HCC: hepatocellular carcinoma
HCCP: paracancer of hepatocellular carcinoma
ICC: intrahepatic cholangiocarcinoma
ICCP: paracancer of intrahepatic cholangiocarcinoma
KEGG: Kyoto Encyclopedia of Genes and Genomes
MALDI: matrix‐assisted laser desorption ionization
MAX: mixed anion‐exchange
MS: mass spectrometry
PSMs: peptide‐spectrum matches
RT: room temperature
TCGA: The Cancer Genome Atlas
TFA: trifluoroacetic acid
TMT: tandem mass tag
WFA: wisteria floribunda agglutinin

## 1 Introduction

Primary liver cancer continues to be a growing health problem worldwide. Hepatocellular carcinoma (HCC) and intrahepatic cholangiocarcinoma (ICC) are two major subtypes of primary liver cancer, accounting for approximately 75–85% and 10–15%, respectively [1, 2]. Both together, they have been the sixth most commonly diagnosed cancer and third leading cause of cancer death globally [2]. Although being a relatively rare cancer, ICC has caused more and more attention with increasing incidence and mortality worldwide [3].

Intrahepatic cholangiocarcinoma arises from the intrahepatic biliary tract and is different from HCC with respect to morphology, metastatic capacity, and their responses to cancer therapy [4]. Distinguishing between ICC and HCC has been a major clinical issue because their management and prognosis differ significantly; however, the molecular pathogenesis underlying these differences is largely unclear [5]. In contrast to the well‐characterized HCC, substantially less is known about ICC. In fact, many ICC patients are actually detected during HCC screening or treatment such as hepatic resection or transplantation for a presumed diagnosis of HCC and such misdiagnosis can then lead to inappropriate use of treatment aimed at HCC [5, 6]. Although more research have been recently reported in terms of genetic, epigenetic, chromosomal, and microRNAs aberrations in ICC [7], the understanding of the molecular abnormalities involved in ICC pathogenesis is still limited. Exploring the molecular alterations underlying the carcinogenesis of ICC as well as the molecular differences between ICC and HCC will be beneficial for the differential diagnosis and targeted clinical treatment of ICC patients.

Glycosylation is one of the most important and complex post‐translational modifications of proteins [8, 9]. It is involved in diverse biological processes, such as recognition and regulatory functions, cell communication, adhesion, immune defense, as well as cell growth and development. Abnormal glycosylation has been recognized as an essential feature of many cancers and plays a fundamental role in key pathological steps of tumor development and progression [10]. In HCC, a number of aberrant glycosylation have been reported [11]. For example, the increased level of α 2,6‐linked sialylation and tetra‐antennary N‐glycans is closely related to HCC progression [12, 13]. In addition, the core‐fucosylated form of alpha fetoprotein (AFP‐L3) has been used as an important serum biomarker for HCC diagnosis [14]. However, only limited lectin‐specific glycans (sWGA‐specific glycans) or glycoproteins (WFA‐Mucin 1 and WFA‐LICAM) have been detected in ICC without detailed glycan structure and glycoprotein information [15, 16, 17]. A systematic analysis is necessary to explore the glycosylation changes in ICC and to discriminate ICC from HCC tumors.

During the past few decades, mass spectrometry‐based glycomic approaches have been widely used for the large‐scale identification of abnormal glycosylation in cancers including HCC [11, 13]. Released N‐glycans in HCC tissues have been widely studied by glycomics based on matrix‐assisted laser desorption ionization imaging mass spectrometry (MALDI‐IMS) [18, 19], MALDI‐TOF‐MS [20], HPLC [13], LC‐MS/MS [21, 22], and DNA sequencer‐assisted carbohydrate electrophoresis [23], while no MS‐based glycomic analysis has been reported in ICC tissues. These glycomic methods could obtain detailed compositions and/or structure analysis of released glycans; however, they lost the glycoprotein and glycosite information. Recently, intact glycopeptide‐based glycoproteomic methods and software have been developed for the simultaneous identification of glycan, corresponding glycosite and glycoprotein. It has also been applied on HCC tissues/cell lines and revealed site‐specific N‐glycan changes in HCC [24, 25, 26]. Unfortunately, most currently used software relies on glycan database for glycopeptide interpretation and mainly provides information on glycan compositions rather than functional structures. Recently, we have developed a novel glycan database‐independent strategy to determine detailed N‐glycan structures on intact glycopeptide through the combined utilization of B and Y ions in tandem mass spectra (MS/MS) with low HCD collision energy [27]. It enables the high‐throughput *de novo* structural sequencing of site‐specific N‐glycans on intact glycopeptide using software termed strucgp [27], which has been applied on the HCC cell lines and revealed site‐specific changes of fucosylated N‐glycans during epithelial–mesenchymal transition of HCC cells [28].

**Fig. 1:**
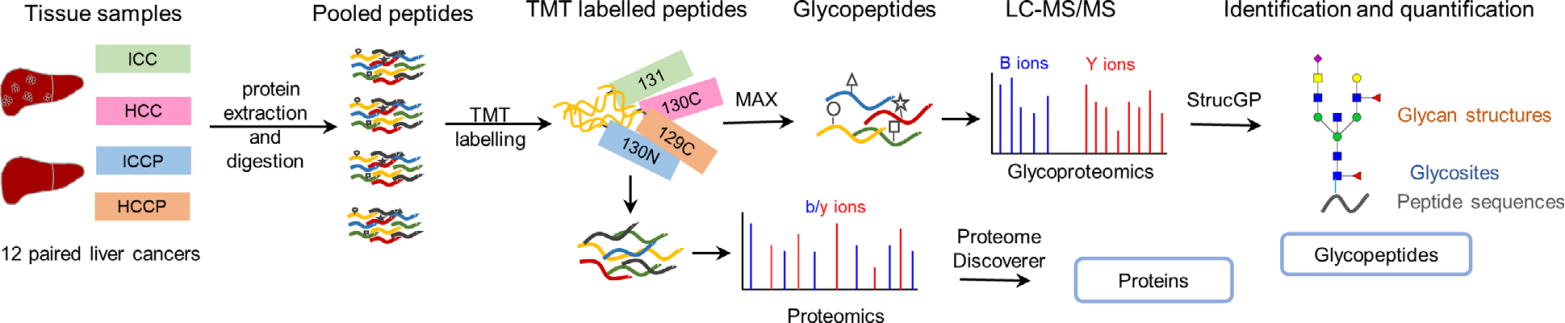
Workflow of quantitative glycoproteomic and proteomic analyses of tissue samples from intrahepatic cholangiocarcinoma (ICC) and hepatocellular carcinoma (HCC). Proteins were extracted from six pairs of tumors and paracancer tissues from ICC and HCC. Equal amounts of tryptic peptides were pooled into four sample groups and labeled with four channels of the TMT reagents. The peptides were combined and separated into two aliquots: one aliquot for intact glycopeptide enrichment and the other aliquot for direct proteomic analysis. Both intact glycopeptides and peptides were analyzed by LC‐MS/MS and quantitatively identified using strucgp and Proteome Discoverer for site‐specific glycosylation and proteomic analysis, respectively.

In this study, we combined the newly developed N‐glycoproteomic methods with stable isotopic labeling‐based quantification and applied them on tumor tissues of ICC and HCC to investigate the differences in aberrant site‐specific N‐glycosylation between ICC and HCC tumors (Fig. 1). The paired paracancer tissues (ICCP and HCCP) were also included as controls to determine the site‐specific glycosylation changes in ICC and HCC tumors. The detailed information of the 12 paired samples is summarized in Table S1. Briefly, the trypsin‐digested peptides of individual tumor/paracancer tissue from the same sample group were pooled into one sample and four pooled samples (including ICC, ICCP, HCC, and HCCP groups) were labeled with the tandem mass tag (TMT) reagents [29]. Glycopeptides were then enriched from TMT‐labeled peptide mixture using mixed anion‐exchange columns (MAX) [30] and analyzed by LC‐MS/MS and strucgp [27], which enables large‐scale and simultaneous identification of N‐glycan structures, N‐glycosites, and modified N‐glycoproteins. By extracting TMT reporter ion intensities from their identified MS/MS spectra, the intact N‐glycopeptides were quantified among four groups. The differences of aberrant site‐specific N‐glycans in ICC and HCC were systematically demonstrated by analyzing their glycan features as well as their modified glycoproteins. The proteomic analysis was also performed to further investigate whether those glycopeptide changes occurred at protein expression or site‐specific glycosylation level. The data will enhance our understanding of glycosylation differences between ICC and HCC that might be associated with their different carcinogenesis and progression.

## 2 Materials and methods

### 2.1 Tissue samples

Tissue samples were prospectively collected from patients undergoing ICC or HCC resection at the First Affiliated Hospital of Xi'an Jiaotong University, China, from 2014 to 2016. The study was approved by Human Ethics Committee at the First Affiliated Hospital of Xi'an Jiaotong University. In total, 12 tumor samples were collected from patients with ICC (*n* = 6) and HCC (*n* = 6), and their paired paracancer tissues (*n* = 12) were also collected as control samples. The age range of the participants was between 20 to 68 years old, with an average age of 54.8. Besides, serum AFP concentrations were negative in all ICC patients and randomly distributed from low to high in HCC patients. The detailed clinical information of samples is summarized in Table S1. All tissue samples were stored at −80 °C until use. All the study methodologies conformed to the standards set by the Declaration of Helsinki.

### 2.2 Protein extraction and digestion

Liver tissues (~ 50 mg tissue per sample) were cut into pieces and washed twice with precooled PBS to remove serum. Then, tissues were suspended with 8 m urea/1 m NH_4_HCO_3_ buffer, homogenized using an automatic rapid tissue homogenizer (60 Hz, Shanghai Jing Xin, China) and sonicated by ultrasonic cell distribution system until the upper solution was clear. The protein concentrations were measured by BCA reagent (Beyotime, Shanghai, China). The proteins were then reduced by 5 mm dithiothreitol (DTT) at 37 °C for 1 h and alkylated by 15 mm iodoacetamide in the dark for 30 min at room temperature (RT), followed by addition of another 2.5 mm DTT for 10 min at RT. Protein pellet solutions were digested overnight by sequencing grade trypsin (Promega, Madison, WI, USA; protein: enzyme, 100 : 1) in 2 m urea/0.25 m NH_4_HCO_3_ buffer at 37 °C. The digested solutions were acidified with trifluoroacetic acid (TFA) to pH < 2 and centrifuged at 15 000 *g* to remove any particulate matter. The peptides were desalted with Oasis HLB column (Waters, Milford, MA, USA) and eluted in 1 mL solution of 60% acetonitrile (ACN)/0.1% TFA. The peptide concentrations were measured by UV absorbance at 215 nm using a DS‐11 spectrophotometer (Denovix, Wilmington, DE, USA).

### 2.3 Tandem mass tag labeling

Equal amounts of tryptic peptides from each tumor or paracancer tissue within the same sample group (six samples per group) were pooled into one sample prior to TMT labeling. Each of four pooled samples (including ICC, ICCP, HCC and HCCP pooled samples) was labeled by one channel of the NH_2_‐reactive N‐terminal labeling reagents (10‐plex TMT; Thermo Fisher Scientific, Waltham, MA, USA) according to the manufacturer's protocols [TMT channel of 129C (TMT‐129C): HCC paracancer; TMT‐130C: ICC paracancer; TMT‐130N: HCC tumor; TMT‐131: ICC tumor]. The concentration of TMT‐labeled peptides was measured by BCA assay. Then, equal amounts of the labeled peptides with four channels were combined and desalted by HLB columns prior to LC‐MS/MS analysis.

### 2.4 Enrichment of N‐glycopeptides using mixed anion‐exchange extraction cartridges

The intact glycopeptides were enriched from TMT‐labeled peptide samples using mixed anion‐exchange columns (MAX, Waters) [30]. Briefly, the tryptic peptide mixtures eluted from HLB column were adjusted to a final concentration of 95% ACN/1% TFA. Peptides were loaded onto activated MAX cartridges twice and washed with 95% ACN/1% TFA for three times. Enriched glycopeptides were eluted in 400 µL solution of 50% ACN/0.1% FA, dried by vacuum concentration with an RVC 2‐18 CDplus concentrator (Christ, Osterode am Harz, Germany), and resuspended in 20 µL of 0.1% FA solution for LC‐MS/MS analysis.

### 2.5 LC‐MS/MS analysis

Each sample underwent triplicate LC‐MS/MS runs on an Orbitrap Fusion Lumos Mass Spectrometer equipped with an Easy‐nLC™ 1200 system (Thermo Fisher Scientific, San Jose, CA, USA). About 1 μg labeled peptide or intact glycopeptide was separated by LC with the use of an Acclaim PepMap100 C18 column (50 cm, 75 μm i.d., 3 μm) protected by a pre‐column (2 cm, 75 μm i.d., 3 μm). The flow rate was kept at 200 nL·min^−1^ with mobile phase consisting of 0.1% FA in water (A) and 0.1% FA in 80% ACN (B).

The gradient profile (110 min) for proteomics was set as follows: 3–7% B for 1 min, 7–35% B for 84 min, 35–68% B for 15 min, 68–100% B for 1 min and equilibrated in 100 B for 9 min. The spray voltage was set at 2.4 kV. Orbitrap MS1 spectra (AGC 4 × 10^5^) were collected from 350–1800 *m/z* at a resolution of 60K followed by data‐dependent HCD MS/MS (resolution 50K, collision energy 37%) using an isolation width of 1.6 Da. Peptides with charge states from 2 to 6 were selected for MS/MS acquisition. A complete gradient profile of 120 min for glycoproteomics was set as follows: 3–7% B for 1 min, 7–35% B for 90 min, 35–68% B for 19 min, 68–100% B for 1 min and equilibrated in 100 B for 9 min. The spray voltage was set at 2.4 kV. Orbitrap MS1 spectra (AGC 4 × 10^5^) were collected from 600 to 1800 *m/z* at a resolution of 60K followed by data‐dependent HCD MS/MS (resolution 50K, collision energy 20% and 37%) of the 20 most abundant ions using an isolation width of 1.6 Da. Peptides with charge states from 3 to 7 were selected for MS/MS acquisition.

### 2.6 Protein identification and quantification

The LC‐MS/MS data of global protein were searched against UniProt human protein databases by Sequest in Proteome Discoverer (Thermo Fisher Scientific, version 2.3) using same parameters described previously [26]. Briefly, the search parameters were set as follows: up to two missed cleavages were allowed for trypsin digestion, 10 p.p.m. and 0.02 Da mass tolerances were set for precursor and MS/MS ions, respectively; carbamidomethylation (C, +57.021464 Da) and 10‐plex TMT (N‐termini of peptides, +229.162932 Da) were set as static modifications; oxidation (M, +15.9949 Da), N‐termini acetylation (+42.010565 Da), and 10‐plex TMT (K, +229.162932 Da) were set as dynamic modifications. All results were filtered with 1% false discovery rate (FDR). The peptides were quantified based on intensities of TMT reporter ions (normalized by total intensities within the TMT set) in their identified MS/MS spectra.

### 2.7 Intact glycopeptide identification and quantification

The identification of intact glycopeptides was performed using strucgp [27]. Briefly, the LC‐MS/MS raw data of glycopeptides were first converted to ‘mzML’ format using Trans‐Proteome Pipline [31] and searched against the built‐in glycan branch structure database from strucgp [27] and UniProt human protein database with the same parameters as mentioned above for proteomic data. The potential glycosite‐containing peptides were screened with the N‐X‐S/T motif (X is any amino acid except Proline). Other parameters were set as follows: at least two oxonium ions out of the top 10 fragment ions in the MS/MS spectra were used for extraction of MS/MS spectra for intact glycopeptide. The mass tolerances of 10 and 20 p.p.m. were allowed for precursors and fragmentation ions. The identification results were filtered with 1% FDR for both peptide sequences and glycan structures, which was estimated by the decoy peptide method [32, 33] and decoy spectra method [27], respectively. The search results were ranked based on their scores, and the peptide/glycan with the highest score was considered to be the corrected identification. In addition to FDR controls at both peptide and glycan levels, a probability strategy was used to further evaluate the reliability of each module of identified glycan structures [27].

The quantification information of intact glycopeptides was extracted from their identified MS/MS spectra based on intensities of TMT reporter ions. The intensity of TMT reporter ions was first normalized using the global scaling factors obtained from global proteomic results to adjust its intensity across the four channels and the normalized intensity was used for quantitative analysis of identified glycopeptide. Quantification data were further filtered with at least five peptide‐spectrum matches (PSMs) per glycopeptide. Median intensities of all PSMs were calculated for each sample and used to calculate the ratios of intact glycopeptide between samples.

### 2.8 Gene ontology and KEGG pathway analyses

The Gene Ontology (GO) and Kyoto Encyclopedia of Genes and Genomes (KEGG) pathway analysis were performed for differentially expressed glycoproteins in ICC and HCC tissue samples using STRING: functional protein association networks (https://string‐db.org). The thresholds of count > 2 and *P*‐value < 0.05 were applied as filters on GO and KEGG pathway analyses of altered glycoproteins.

### 2.9 Other bioinformatic analysis

Multivariate statistic principal component analysis was created in R 3.2.2 using the factominer (http://CRAN.R‐project.org/) [34] for four groups of samples (tumor and paracancer tissues of ICC and HCC) based on their intact glycopeptide expression profiles. For two‐way hierarchical clustering analysis, log2 fold change of abundance ratios of differentially expressed glycopeptides among four different groups was clustered using the pheatmap package in R language using Euclidean as a distance measure for column and row clustering.

### 2.10 Fluorescence‐based lectin histochemistry and immunohistochemistry

Formalin‐fixed paraffin‐embedded (FFPE) tissue sections from the paired tumor and paracancer tissue of ICC were dewaxed and hydrated with xylene and gradient alcohol, respectively. For lectin histochemistry, Cy5‐labeled WFA was applied to detect the LacdiNAc structures according to previously described protocols [35] with some modifications. Briefly, the sections were washed, blocked with 5% (w/v) BSA and 0.04% Triton for 1 h and incubated with 1 μg·μL^−1^ Cy5‐labeled WFA overnight in the dark at RT, followed by staining with DAPI (1 μg·mL^−1^ in PBS; Solarbio, Beijing, China). The fluorescence images were acquired by a fluorescence microscopy, and the relative intensity of fluorescence was quantified by imagej software (http://imagej.net).

For immunohistochemistry, the pretreated FFPE tissue sections were blocked and incubated with anti‐B4GALNT3 antibody (orb31745l Biorbyt, UK) or anti‐B4GALNT4 antibody (orb546266; Biorbyt, Cambridgeshire, UK) at a 1 : 100 dilution, followed by detection using the PV and DAB chromogenic kits (Servicebio, Wuhan, China). The slides were scanned using Leica SCN400 slide scanner (Leica Biosystems, Wetzlar, Germany), and five different fields of view were randomly selected in each section for quantification.

### 2.11 Gene expression analyses in public datasets

The transcriptome profiling data and clinical data were obtained from The Cancer Genome Atlas (TCGA) Data Portal (https://tcga‐data.nci.nih.gov/tcga/tcgaHome2.jsp). The expression matrix of B4GALNT3 and B4GALNT4 in ICC and HCC patient tissues were obtained from the gene expression of RNAseq (HTSeq‐Counts) dataset for cholangiocarcinoma (CHOL) [36] and liver hepatocellular carcinoma (LIHC) [37]. In order to exclude other cancers, samples belong to ICC and HCC were further extracted from the CHOL and LIHC datasets according to the primary pathology classification in clinical information. There are 41 samples available for ICC (33 tumors and 8 normal tissues) and 413 samples for HCC (364 tumors and 49 normal tissues). The raw data of gene expression levels were log_2_(*x* + 1)‐transformed and processed using deseq2 package in R language. Statistical significance of the expression differences between groups was determined using Wilcox's test.

## 3 Results and Discussion

### 3.1 Structural profiling of site‐specific N‐glycans in ICC and HCC

By using our recently developed glycoproteomic method and glycan database‐independent software strucgp, a number of 4741 unique intact N‐glycopeptides were identified within 1% false discover rate (FDR) from TMT‐labeled tumor and paracancer tissue samples of ICC and HCC (Table S2). These intact glycopeptides consist of 486 N‐glycans structures (221 compositions) modified at 1235 glycosite‐containing peptides from 894 N‐glycoproteins.

**Fig. 2:**
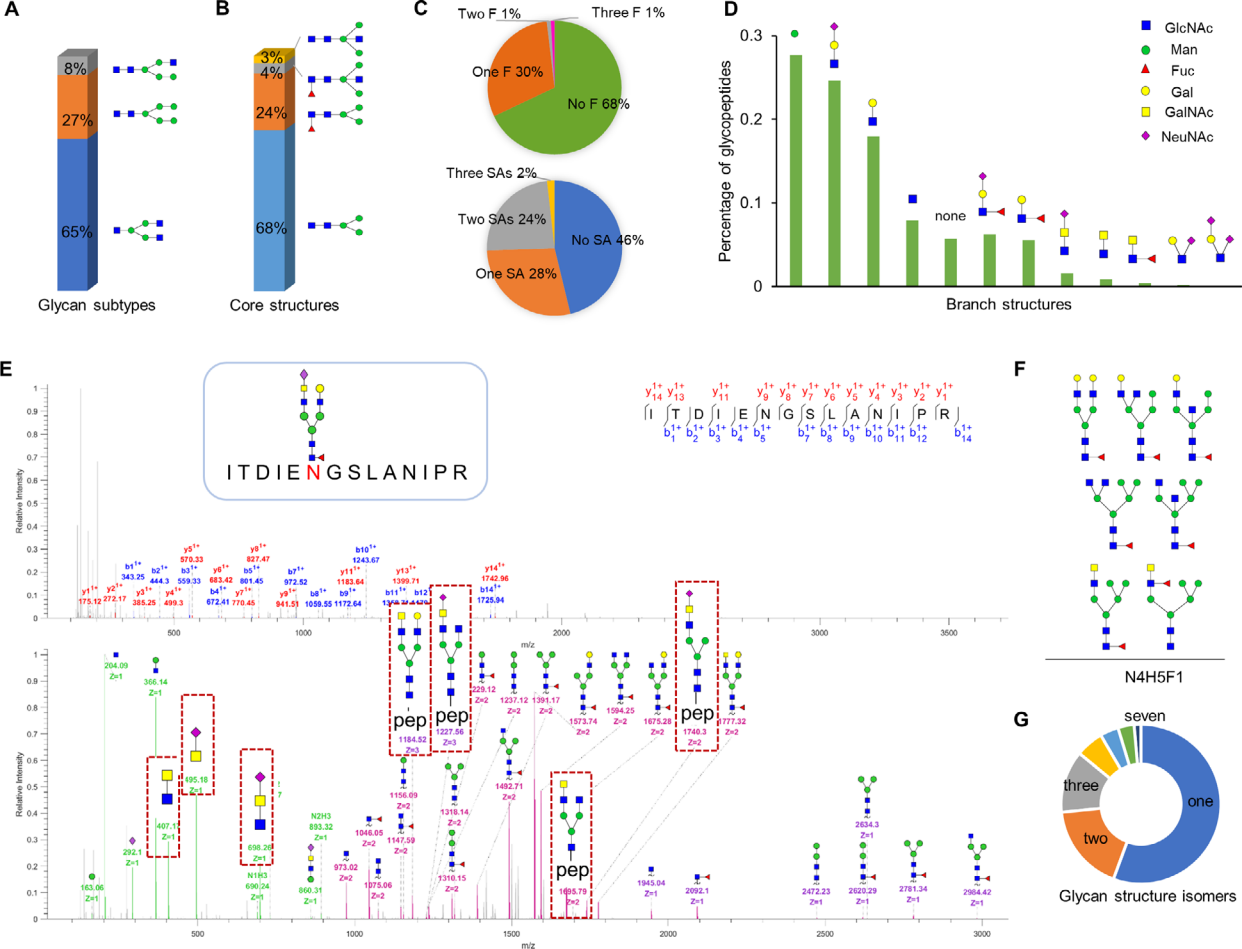
Large‐scale profiling of intact glycopeptides in ICC and HCC samples (*n* = 24). (A) Proportions of different glycans based on glycan subtypes. (B) Proportions of four types of core structures in ICC and HCC. (C) Intact glycopeptide PSMs of different glycans based on the numbers of fucose and sialic acid modifications. PSM, peptide‐spectrum matches; F, fucose; SA, sialic acid. (D) Twelve branch structures identified in ICC and HCC and their relative abundances. (E) Representative MS/MS spectra for identification of LacdiNAc‐containing glycopeptide: peptide ITDIE^#^NGSLANIPR modified by a LacdiNAc‐containing glycan (N5H4F1S1) from Asporin (ASPN). ^#^indicates the glycosylation site. The sequence of peptide was identified by matched b and y ions (labeled in blue and red, respectively) in MS/MS spectrum with high HCD energy (HCD = 37, upper spectrum). The glycan structure was identified by B and Y ions (labeled in green and purple, respectively) in MS/MS spectrum with low HCD energy (HCD = 20, bottom spectrum). (F) Seven different glycan structures discriminated from one glycan composition (N4H5F1). (G) Number of glycan structure isomers identified in ICC and HCC tissues.

Based on module structures of the glycans, the identified N‐glycans in ICC and HCC were composed of four types of core structures and 12 branch structures with three glycan subtypes. In terms of glycan subtypes, more than 65% of glycosites were occupied by complex glycans, followed by oligo‐mannose glycans (27%), and hybrid glycans (Fig. 2A). Among the core structures, the typical core structure HexNAc2Hex3 accounted for 68% of all unique glycopeptides, followed by fucosylated core structure (24%) and bisecting core structure (Fig. 2B). In addition, about one‐third of the glycosites (32%) were occupied by fucosylated glycans with one to three fucoses (Fig. 2C) and 92.7% of identified fucosylated glycans were assigned as core‐fucosylated, which indicated a general occurrence of core fucose in ICC and HCC tissues. More than half of glycosites (53.8%) were modified by sialylated glycans containing 1–3 sialic acids (Fig. 2C). The percentages of fucosylation and sialylation identified in this study were consistent with previously reported glycoproteomic results in HCC [26]. Among the 12 identified branch structures, the common LacNAc‐containing (HexNAc + Hex) branches accounted for most of intact glycopeptides: LacNAc with or without Neu5AC took 43%, LacNAc with or without fucose took 12% (Fig. 2D). Interestingly, the uncommon LacdiNAc‐containing (HexNAc + HexNAc) branches with or without Neu5Ac/fucose were also identified by characteristic B ions containing two HexNAc (HexNAc2, HexNAc2Sia1, or HexNAc2Fuc1) (Fig. 2D). The characteristic B ions have already been used for identification of LacdiNAc‐containing structures on purified glycoproteins such as prostate‐specific antigen [38] and the SARS‐CoV‐2 S protein [39]. However, no direct identification of LacdiNAc structures has been reported in previous glycomic or glycoproteomic analysis of HCC/ICC tissues/cell lines [18, 19, 20, 21, 22, 23, 24, 25, 26].

The identification of LacdiNAc‐containing branches further leads to identification of LacdiNAc‐containing glycopeptides by characteristic B ions and corresponding Y ions with low energy HCD fragmentation. Representative MS/MS spectra in Fig. 2E shows the identification of glycopeptide with sialylated LacdiNAc by a series of feature B ions (HexNAc2 ion at *m/z* 407.17, HexNAc2Sia1 at *m/z* 698.26, HexNAcSia1 at *m/z* 495.18) and Y ions (such as peptide+HexNAc4Hex3Fuc1Sia1 at *m/z* 1740.3, peptide+HexNAc5Hex4Fuc1 ion at *m/z* 1184.52, and peptide+HexNAc5Hex3Fuc1Sia1 at *m/z* 1227.56). Among the 486 identified N‐glycans, up to seven different glycan structure isoforms can be discriminated from one single composition (Fig. 2F) and nearly half of the glycan compositions could be sorted into at least two distinct glycan isoforms (Fig. 2G). Furthermore, some glycan isoforms attached at the same peptide (but not all) could be directly separated by the C18 column. For example, different isomers of the glycan with a composition of HexNAc5Hex4Fuc1 could be eluted at separated retention time in the LC–MS (Fig. S1). Overall, structural characterization of site‐specific N‐glycans has great potential to identify novel aberrant glycosylation in ICC and HCC.

### 3.2 ICC is different from HCC tumors at both glycoprotein and site‐specific glycosylation levels

Based on intensities of TMT reporter ions, the intact N‐glycopeptide abundances among ICC, HCC, and their paired paracancer tissues were quantified and compared. The TMT‐based glycoproteomic enables the quantification of glycopeptides with isomeric N‐glycans by directly extracting the abundances of TMT reporter ions from the MS2 spectra of identified glycopeptides. To improve the quantification accuracy, only glycopeptides with at least five PSMs were selected, which resulted in quantification of 1084 glycopeptides from 212 glycoproteins (Table S3).

**Fig. 3:**
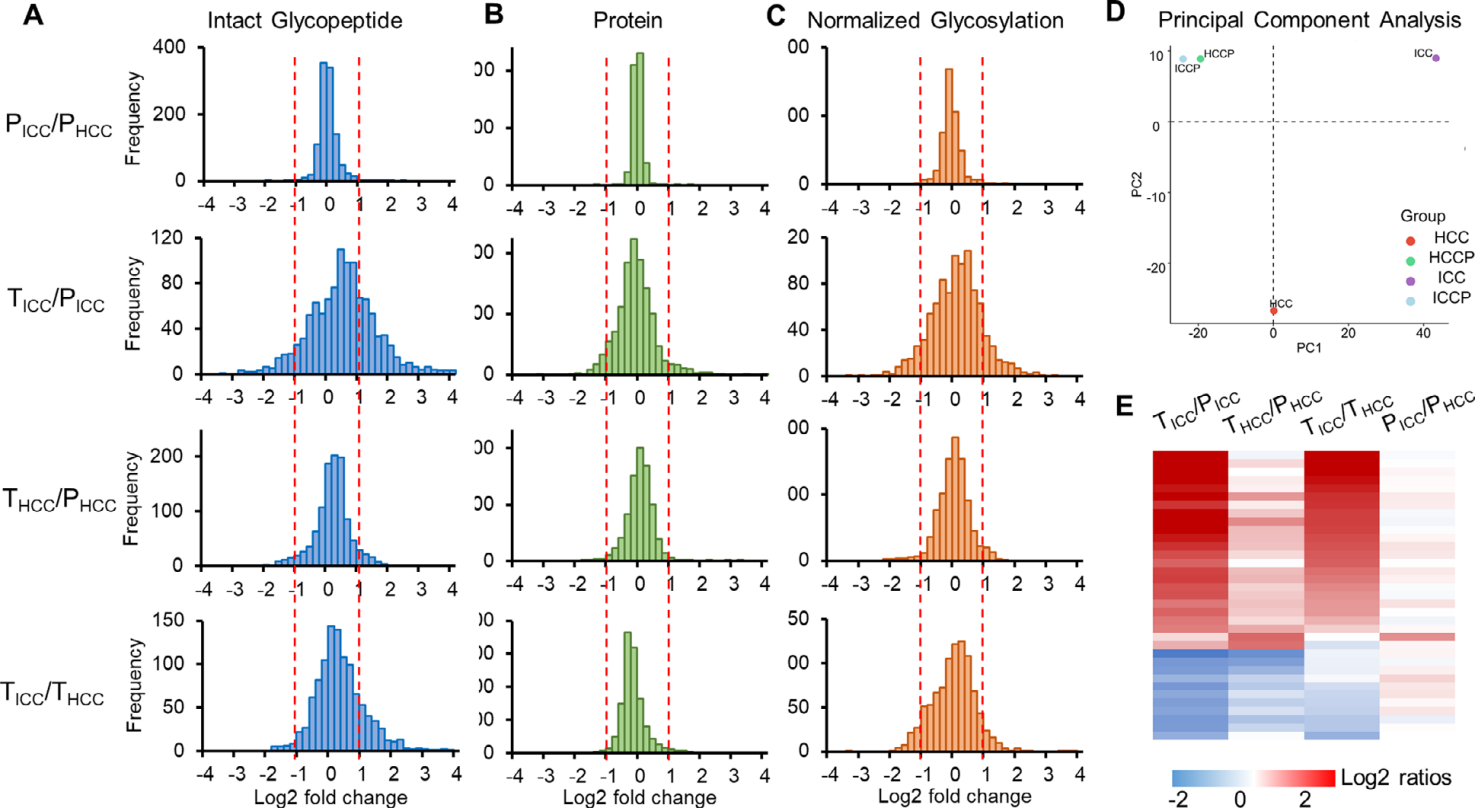
Relative quantification of intact glycopeptides, proteins, and glycosylation occupancies among different sample groups (*n* = 24). (A–C) The frequencies of intact glycopeptides (A), proteins (B), and glycosylation occupancies (C) were compared between paracancer tissues of ICC and HCC (upper, PICC/PHCC), ICC tumors and paired paracancer tissues (TICC/PICC), HCC tumors and paired paracancer tissues (THCC/PHCC), as well as ICC and HCC tumors (TICC/THCC). (D) Principal component analysis of intact glycopeptides identified from ICC and HCC tumors as well as their paired paracancer tissues (ICCP and HCCP). (E) Heatmap of altered glycoproteins in ICC or HCC tumors compared with their paired paracancer tissues at protein expression level.

We first compared the glycopeptide abundances between two groups of paracancer tissues, which indicated that over 98% of N‐glycopeptides were within twofold changes (Fig. 3A). By using twofold change as a cutoff, we found that when compared with paired paracancer samples, 37.9% and 10.6% of N‐glycopeptides changed in ICC and HCC, respectively. A direct comparison between ICC and HCC showed that 21.1% of glycopeptides changed between ICC and HCC (Fig. 3A). These results indicated that differences of glycopeptides existed not only between tumors (ICC and HCC) and their paired paracancer tissues, but also between ICC and HCC tumors. This observation was further confirmed by the principal component analysis of intact glycopeptides among four groups, which indicated that ICC tumors, HCC tumors, and their paracancer tissues are located in three distinct regions (Fig. 3D).

At the global proteome level, 10% and 2.2% of 2545 quantified proteins varied more than twofold in ICC and HCC tumors in comparison with their paired paracancer tissues, respectively (Fig. 3B). A comparison between ICC and HCC showed that 3.6% of proteins varied more than twofold. The results indicated a smaller degree of change at proteomic level. By focusing on glycoproteins identified in both glycoproteomic and proteomic data, we found that 36 glycoproteins were altered (≥ 2‐fold change) in ICC and/or HCC compared with their paired paracancer tissues. By excluding any glycoprotein that changed between two groups of paracancers, a further difference (≥ 2‐fold change) of 17 glycoproteins was observed between ICC and HCC tumors including 16 up‐regulated and one down‐regulated glycoproteins in ICC (Fig. 3E and Table S4). GO analysis on these 17 altered N‐glycoproteins showed that they were mainly located in extracellular matrix region, followed by Golgi apparatus part, lysosomal lumen, and endoplasmic reticulum part (Fig. S2), which is consistent with the normal distribution of N‐glycoproteins. KEGG analysis showed that they were mainly enriched in pathways including proteoglycans in cancer, TGF‐beta signaling pathway, ECM–receptor interaction, and focal adhesion (Fig. S2). All those pathways are important cellular processes regulating cell proliferation, adhesion and survival and the abnormalities of them are closely connected to various types of cancers [40, 41, 42].

To determine whether these glycopeptide changes occurred at the protein or glycosylation levels, we calculated normalized glycosylation by dividing intact glycopeptide ratios with their glycoprotein ratios. Using the approach, we found that the normalized glycosylation on 20.1% and 6.2% of glycopeptides changed more than twofold in ICC and HCC tumors compared with their paired paracancer tissues, respectively (Fig. 3C). The comparison between ICC and HCC tumor samples showed that 13.1% of glycopeptides changed at glycosylation level. These results indicated that the site‐specific glycosylation changes in ICC were much greater than that of HCC. Altogether, these data indicated a huge distinction between ICC and HCC tumors at both glycoprotein and site‐specific glycosylation levels.

### 3.3 Bi‐antennary and bisecting glycans are commonly increased in ICC and HCC tumors

**Fig. 4:**
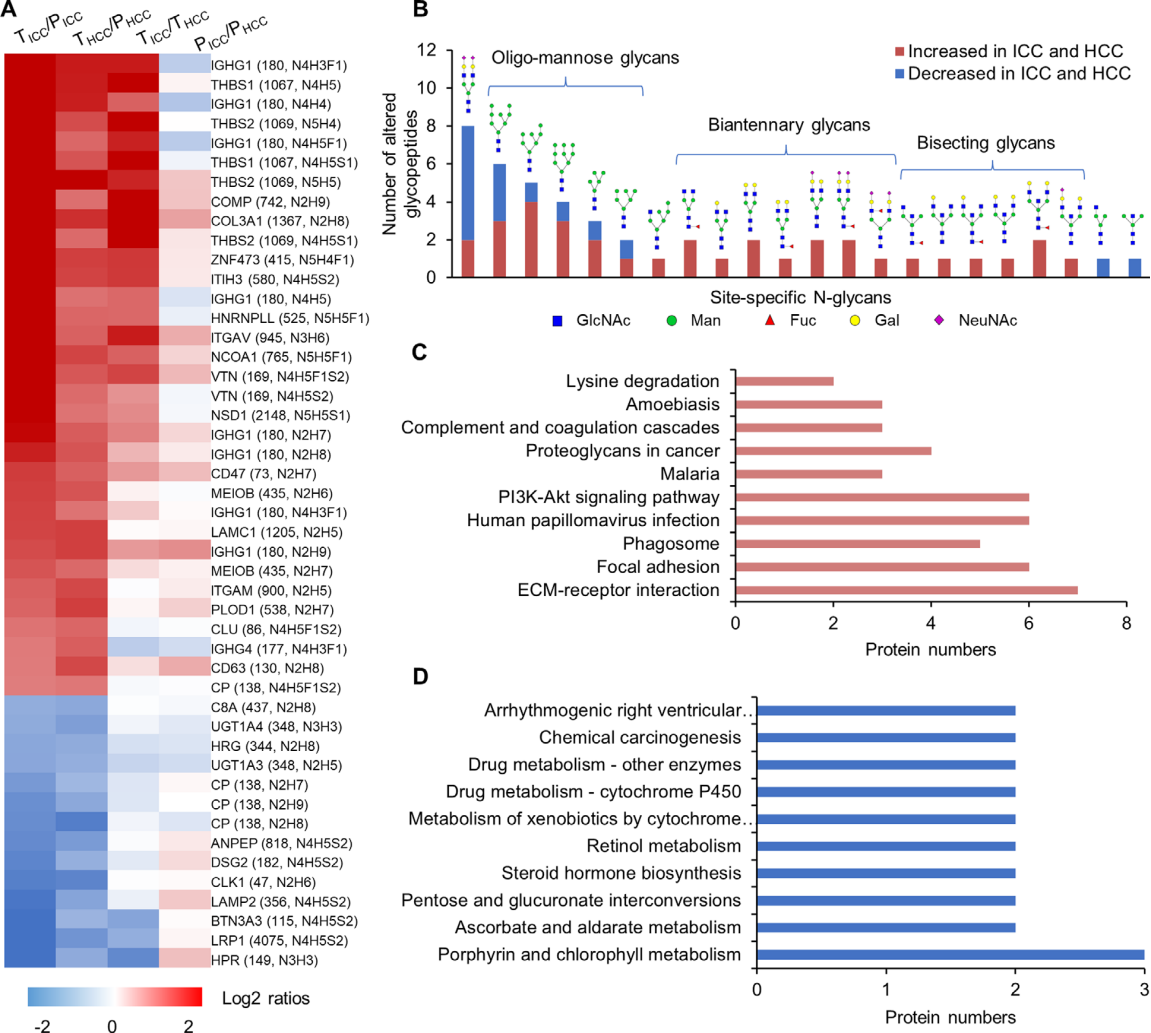
Glycopeptides commonly altered in both ICC and HCC tumors compared with paracancer tissues. (A) Heatmap of 33 increased and 14 decreased glycopeptides in both ICC (*n* = 6) and HCC (*n* = 6) tumors. The log2 ratios between groups were calculated based on the normalized abundance of TMT labeling. T_ICC_, tumors of ICC; P_ICC_, paracancers of ICC; T_HCC_, tumors of HCC; P_HCC_, paracancers of HCC. (B) Frequencies of commonly changed site‐specific glycans in ICC (*n* = 6) and HCC (*n* = 6) tumors. (C, D) The KEGG pathways enriched in the corresponding glycoproteins of commonly increased (C) or decreased (D) glycopeptides in ICC and HCC tumors.

To investigate the differences of site‐specific glycan occurred at glycosylation level, we first focused on glycopeptides that were commonly changed in both ICC and HCC tumors compared with paired paracancerous tissues. These changes largely represented the common changes of site‐specific glycosylation in the tumorigenesis of both ICC and HCC tumors. By excluding the glycopeptides that changed only at protein expression level, we totally identified 47 glycopeptides with at least twofold changes compared with their paracancers, including 33 increased (up to 20‐fold increase) and 14 decreased glycopeptides (up to 10‐fold decrease) (Fig. 4A and Table S5). These glycopeptides were comprised of 22 N‐glycans and 31 glycosites from 32 glycoproteins.

By classifying these changed site‐specific glycans based on their attached glycan structures, we found that 13 intact glycopeptides modified by bi‐antennary glycans with 0–2 sialic acid, seven glycopeptides modified by bisecting glycans, and 13 glycopeptides modified with oligo‐mannose glycans (Man5 to Man9) were increased in both ICC and HCC (Fig. 4B). Their quantification further demonstrated that the greatest alteration occurred in glycopeptides containing bi‐antennary and bisecting glycans with 5‐ to 20‐fold changes. These increased glycopeptides were identified from 21 glycoproteins, which were mainly involved in pathways of ECM–receptor interaction, focal adhesion, PI3K‐Akt signaling pathway, proteoglycans in cancer, and complement and coagulation cascades (Fig. 4C). As a key regulator involved in cell growth, motility and survival, the aberrant activation of PI3K‐Akt signaling pathway could promote the survival and proliferation of tumor cells in many cancers [43]. As an important component of the inflammatory responses, complement and coagulation cascades is involved in tumors including HCC [26]. In addition, the bi‐antennary glycans with two sialic acids but no fucose (N4H5S2) and oligo‐mannose glycans were also observed in decreased glycans in ICC and HCC (Fig. 4B). These decreased glycopeptides were identified from 12 glycoproteins and three of them (UGT1A3, UGT1A4, CP) were enriched in KEGG pathways including porphyrin and chlorophyll metabolism, ascorbate and aldarate metabolism, retinol metabolism, and drug metabolism (Fig. 4D). GUT1A3 and UGT1A4 were UDP‐glucuronosyltransferases that transforms small lipophilic molecules onto water‐soluble metabolites.

Although those glycopeptides were commonly changed in both tumor groups compared with their paired paracancer tissues, varying degrees of change were observed between ICC and HCC. Among the 47 commonly increased glycopeptides, 21 glycopeptides showed additional differences (≥ 2‐fold change) between ICC and HCC but no significant differences were observed between the two groups of their paracancer tissues (Fig. S3). Those changes were resulted from a greater degree of increase or decrease of glycosylation in ICC than HCC when compared with their paired paracancers.

### 3.4 LacdiNAc‐containing glycans, tri‐antennary glycans, and core‐fucosylated glycans are specifically elevated in ICC tumors

We next studied on the intact glycopeptides that show differential expressions between ICC and HCC tumors (≥ 2‐fold change) but without significant differences between their paracancer tissues. By excluding the glycopeptides that changed only at protein expression level, we totally identified 95 glycopeptides that were specifically altered (up to 19‐fold increase and sixfold decrease) in ICC or HCC tumors compared with their paired paracancers (Fig. S4 and Table S6). These glycopeptides showed additional twofold changes between ICC and HCC tumors and therefore should be specific in two groups of tumors and associated with the differences between ICC and HCC tumors.

**Fig. 5:**
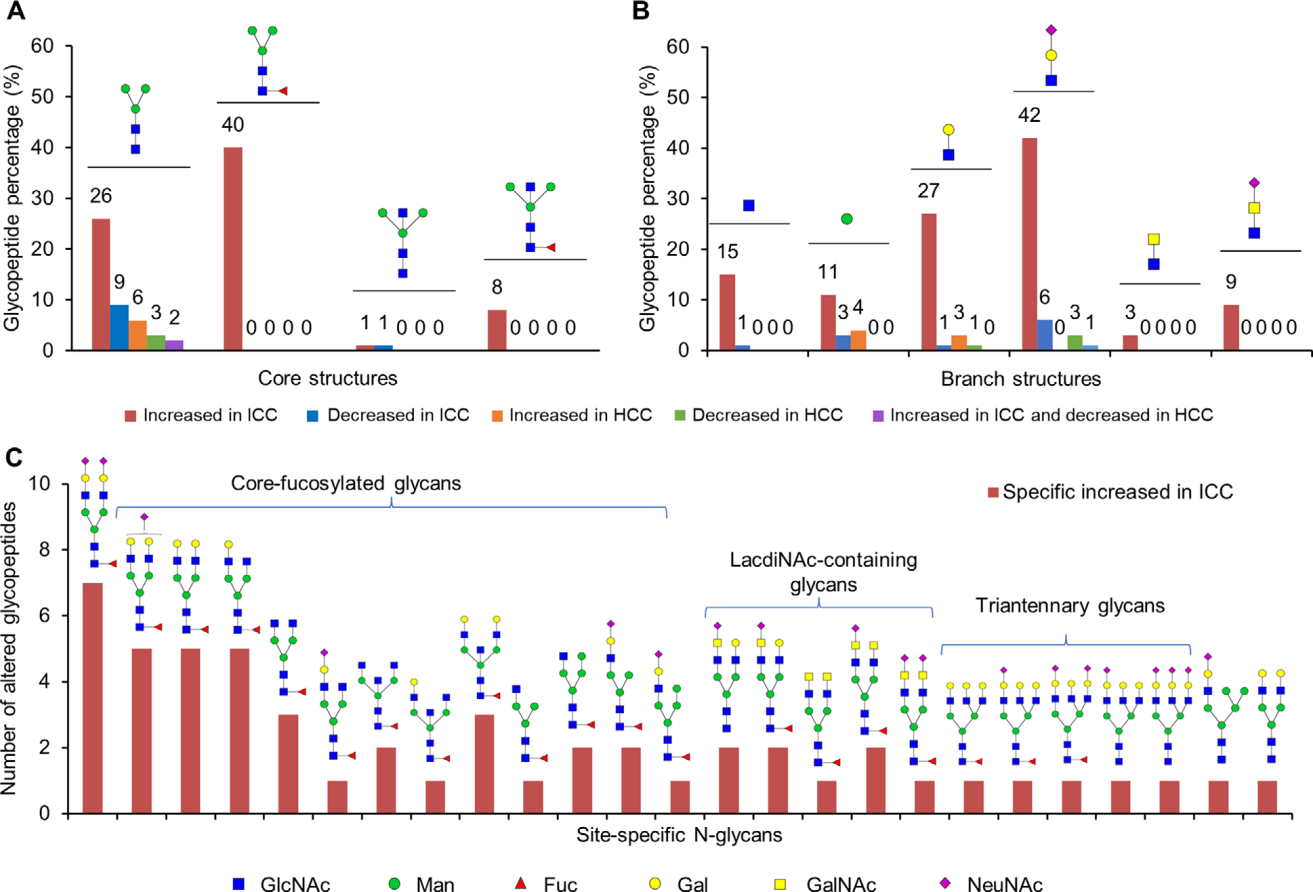
Glycopeptides with altered site‐specific glycosylation in either ICC or HCC tumor. (A) Proportions of four types of core structures that uniquely altered in ICC (*n* = 6) and/or HCC (*n* = 6) tumors: solely increased in ICC (in red), solely decreased in ICC (in blue), solely increased in HCC (in orange), solely decreased in HCC (in green), increased in ICC and decreased in HCC (in purple). (B) Identified branch structures and their proportions in the five groups of uniquely altered N‐glycopeptides. (C) Frequencies of site‐specific glycans that uniquely altered in ICC tumors.

Compared with their paired paracancer tissues, the majority of these glycopeptides (84/95, 88.4%) were specifically altered in ICC (with 2‐ to 19‐fold changes), followed by nine glycopeptides specifically altered in HCC (with twofold to fourfold changes) and two glycopeptides with opposite changes in ICC and HCC (Figs S4 and S5). By classifying these glycans based on their core structures, we found that the fucosylated core structure and core structure with both core fucose and bisecting HexNAc were specifically increased in ICC (Fig. 5A). Moreover, among the six identified branch structures, LacNAc (with or without Neu5Ac) took the majority (69%) of glycopeptides increased in ICC, while LacdiNAc (with or without Neu5Ac) were solely observed in site‐specific glycans increased in ICC (Fig. 5B).

Based on the whole glycan structures, we found that eight glycopeptides modified by LacdiNAc‐containing (GalNAc*β*1–4GlcNAc) glycans and four glycopeptides modified with tri‐antennary glycans with or without Neu5Ac/fucose were specifically increased in ICC (Fig. 5C). In addition, 47 of those specifically increased glycans in ICC were core‐fucosylated. All together, these results indicated that increased glycosylation by core‐fucosylated glycans, LacdiNAc‐containing glycans, and tri‐antennary glycans were uniquely occurred in ICC. The increased levels of core fucosylation and branching are closely related to progression of many cancers including HCC [44]; however, increased core fucosylation does not coincide with increased branches in tissues of HCC patient [13]. According to our previously reported results, the core‐fucosylated glycans were uniquely increased in HCC with high AFP level while the multi‐branched glycans were uniquely increased in HCC with low AFP level [26]. Those increased glycans were identified from 28 glycoproteins, which were involved in pathways of ECM–receptor interaction, complement and coagulation cascades, focal adhesion, proteoglycans in cancer, and PI3K‐Akt signaling pathway (Fig. S6). It can be seen that similar pathways were enriched as those commonly altered glycoproteins described above since several key glycoproteins (ITGAV, THBS1, THBS2, VTN, CLU) were modified by both commonly altered glycans and specifically increased glycans.

### 3.5 Almost all LacdiNAc‐containing N‐glycans are specifically up‐regulated in ICC tumors

**Fig. 6:**
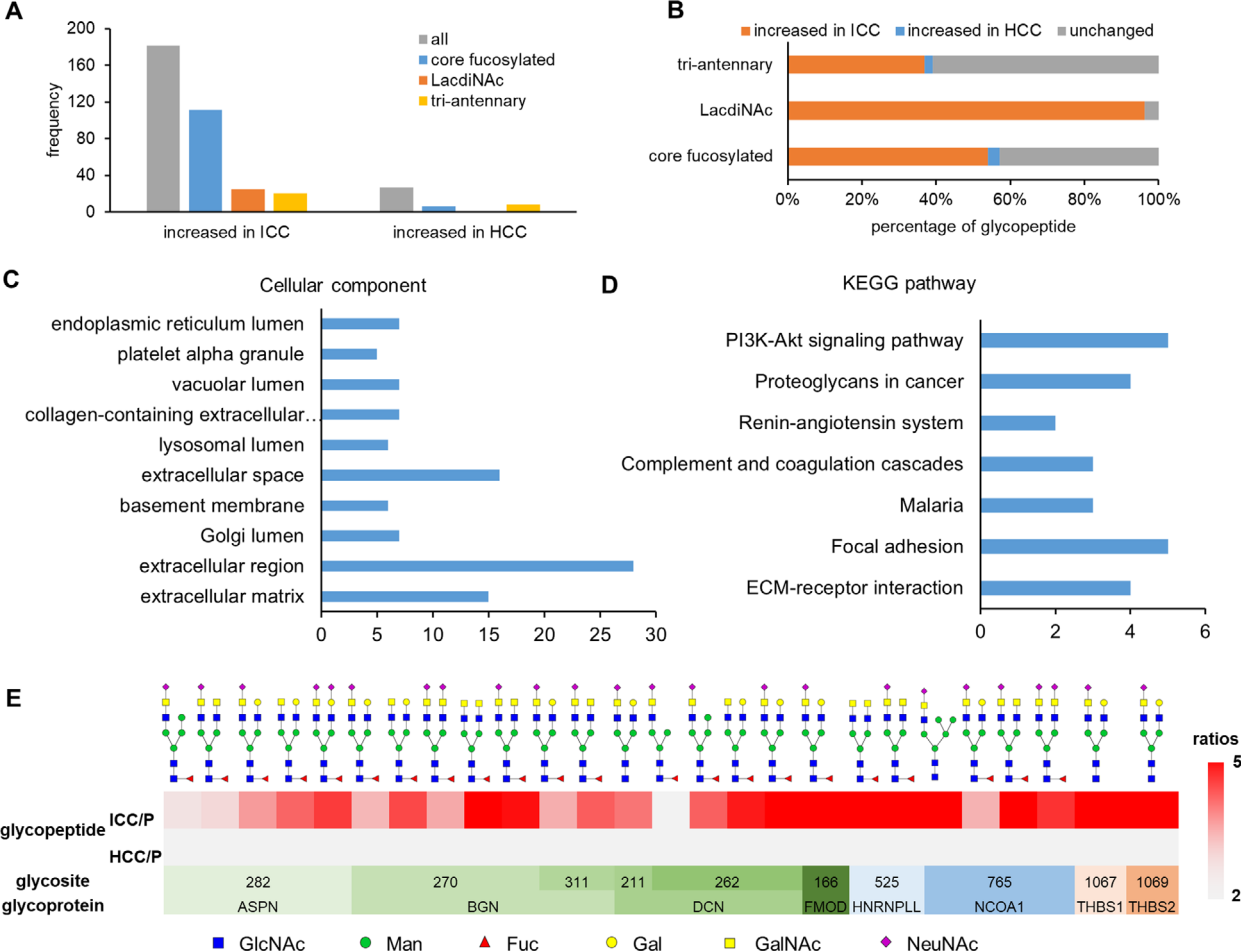
Specific increase of LacdiNAc structures in ICC tumors (*n* = 6). (A) Frequencies of core‐fucosylated glycans, LacdiNAc‐containing glycans, and tri‐antennary glycans uniquely increased in ICC or HCC tumors. (B) Percentages of altered site‐specific glycans in ICC or HCC for core‐fucosylated, LacdiNAc‐containing, and tri‐antennary glycans. (C, D) Enriched cellular components (C), and KEGG pathways (D) of the 63 LacdiNAc‐containing glycoproteins in ICC and HCC tumors. (E) Heat map of all quantitative LacdiNAc‐containing N‐glycopeptides showing the fold changes between tumor (ICC or HCC) with paracancer tissues.

To further evaluate the alteration specificities of those glycans in ICC, we investigated all the altered glycopeptides in ICC or HCC at the glycopeptide level. It indicated that altered glycopeptides modified with core‐fucosylated glycans or tri‐antennary glycans were detected in both ICC and HCC, while all altered glycopeptides with LacdiNAc structures were solely detected in ICC (Fig. 6A). Moreover, among all quantified glycopeptides modified with those glycans, only 57% of core‐fucosylated glycans and 39% of tri‐antennary glycans were found to be increased in ICC or HCC, while almost all of LacdiNAc‐containing glycans (96%) were found to be increased in ICC (Fig. 6B). Overall, the results suggested the increase of LacdiNAc‐containing N‐glycans is very specific in ICC tumors.

LacdiNAc structures are rarely observed in normal mammalian cells; however, their expressions (evaluated by lectins or glycotransferases) were found to be significantly elevated in tumor tissues of serous ovarian [45, 46], peritoneal cancers [47], as well as ICC [48]. Recently, the LacdiNAc‐glycosylated prostate‐specific antigen was identified as potential serum biomarker for prostate cancer [49]. Despite their importance, their functions are largely unknown mainly limited by available analytical techniques for direct analysis of LacdiNAc structures. By using our newly developed structural characterization software strucgp, we were able to discriminate the LacdiNAc‐containing N‐glycans from other glycan isomers at the glycopeptide level. A total of 36 N‐glycans with solely LacdiNAc (GalNAcβ1‐4GlcNAc), sialylated LacdiNAc (Neu5Acα2‐6GalNAcβ1‐4GlcNAc), and fucosylated LacdiNAc (GalNAcβ1‐4GlcNAcα1‐3/4Fuc) structures were identified in ICC and HCC. Those LacdiNAc‐containing N‐glycans were modified on 113 glycopeptides from 33 glycoproteins and were mainly located in extracellular region especially in extracellular matrix, followed by Golgi apparatus part, lysosomal lumen, and endoplasmic reticulum part (Fig. 6C). KEGG analysis showed that they were mainly enriched in ECM‐receptor interaction, focal adhesion, complement and coagulation cascades, proteoglycans in cancer, and PI3K‐Akt signaling pathway (Fig. 6D). The cellular component and KEGG analysis showed similar results with that enriched from other glycoproteins as described above, indicating a relative conservative distribution of altered N‐glycoproteins in ICC and HCC.

By excluding glycopeptides with less than five PSMs, 26 LacdiNAc‐containing glycopeptides were reliably quantified in ICC and HCC. Those glycopeptides comprised of 10 LacdiNAc‐containing N‐glycans modified at 10 glycosites from eight glycoproteins (ASPN, BGN, DCN, FMOD, HNRNPLL, NCOA1, THBS1, and THBS2) (Fig. 6E and Table 1). Among them, small leucine‐rich proteoglycans (ASPN, BGN, DCN, FMOD) and thrombospondin family proteins (THBS1 and THBS2) have been linked to several tumors including gastric, colon, and bladder cancers [50, 51]. NCOA1 is known to promote metastasis and angiogenesis in breast cancer [52], while HNPNPLL is able to suppress metastasis in colorectal cancer [53]. Furthermore, THBS2 has been proposed as novel diagnostic biomarker for early distal cholangiocarcinoma [54]. However, the glycosylation on them has not been fully explored. In this study, TMT‐based quantification of those glycopeptides revealed that 25 out of the LacdiNAc‐containing glycopeptides were elevated in ICC tumor (twofold to fivefold changes) compared with their paired paracancer tissues at the glycopeptide level, while no significant changes were observed in HCC tumor (Fig. 6E). These results not only for the first time confirmed the modification of LacdiNAc‐containing N‐glycans on these glycoproteins, but also revealed a highly specific alteration of LacdiNAc structures in ICC. This suggested important and specific roles of LacdiNAc‐containing glycans and corresponding proteins in ICC, even though their function implications still need further investigations. More importantly, these glycans/glycoproteins/glycopeptides with specific glycosylation alteration have great potential to serve as diagnostic biomarkers or specific targets for therapeutic intervention.

**Table 1:** All quantified site‐specific glycans with LacdiNAc structures in ICC and HCC. 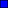: GlcNAc; 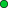: Man; 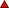: Fuc; 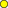: Gal; 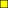: GalNAc; 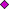: NeuNAc.

No.	Glycoprotein	Gene name	Peptide sequence	Glycan structure	Ratio of glycopeptides			
					ICC/ICCP	HCC/HCCP	ICC/HCC	ICCP/HCCP
1	Asporin	ASPN	ITDIE^282^N^#^GSLANIPR	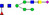	2.21	1.30	1.56	0.92
2				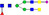	2.33	1.39	1.69	1.01
3				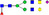	3.08	1.46	2.00	0.94
4				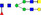	3.75	1.50	2.30	0.92
5				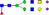	4.28	1.80	3.04	1.28
6	Biglycan	BGN	MIE^270^N^#^GSLSFLPTLR	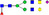	2.73	1.42	2.09	1.08
7				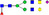	2.85	1.17	2.25	0.93
8				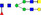	4.11	1.44	2.51	0.88
9				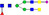	3.86	1.29	2.63	0.88
10				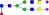	2.91	1.09	2.76	1.04
11			LLQVVYLHSN^311^N^#^ITK	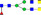	6.02	1.77	2.89	0.85
12				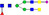	4.83	1.46	3.11	0.95
13	Decorin	DCN	LGLSFNSISAVD^262^N^#^GSLANTPHLR	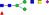	1.55	0.78	1.62	0.82
14				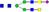	3.55	1.17	2.62	0.86
15				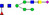	3.81	1.14	2.89	0.86
16				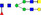	4.69	1.33	2.95	0.83
17			IADT^211^N^#^ITSIPQGLPPSLTELHLDGNK	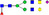	6.62	1.47	4.00	0.89
18	Fibromodulin	FMOD	LYLDHN^166^N^#^LTR	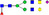	7.39	1.75	4.39	1.04
19	Heterogeneous nuclear ribonucleoprotein L‐like	HNRNPLL	VP^525^N^#^GSNPYTLK	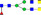	14.87	1.67	9.85	1.11
20				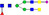	10.94	1.62	7.42	1.10
21	Nuclear receptor coactivator 1	NCOA1	STP^765^N^#^LSLDDVK	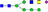	6.33	1.80	3.53	1.00
22				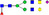	2.80	1.22	2.54	1.11
23				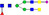	7.84	1.32	6.01	1.01
24				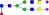	4.39	1.37	4.14	1.29
25	Thrombospondin‐1	THBS1	VV^1067^N^#^STTGPGEHLR	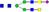	9.29	1.86	6.69	1.34
26	Thrombospondin‐2	THBS2	VV^1069^N^#^STTGTGEHLR	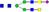	11.44	1.95	6.63	1.13

### 3.6 Confirmation of increased LacdiNAc glycans in ICC tumors

**Fig. 7:**
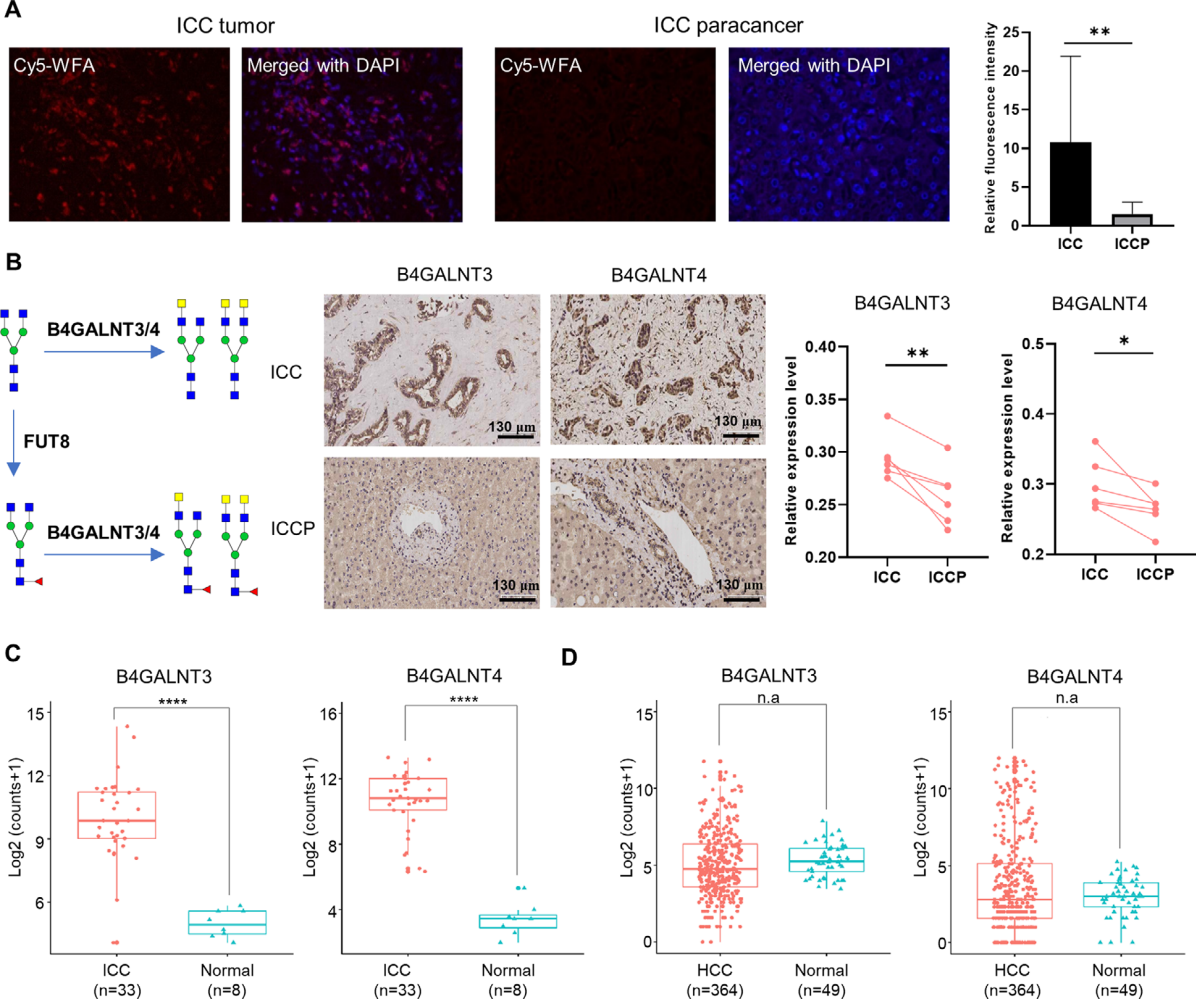
Confirmation of the increase of LacdiNAc‐containing glycans in ICC tumors. (A) Lectin histochemistry analysis of ICC tumors and paired paracancer tissues (*n* = 6, ±SD, *P* = 0.0012 by paired *t*‐test) based on the WFA binding (40× magnification). (B) Immunohistochemistry analysis on the protein expressions of B4GALNT3 (*n* = 6, ±SD, *P* = 0.0061 by paired *t*‐test) and B4GALNT4 (*n* = 6, ±SD, *P* = 0.0341 by paired *t*‐test) from ICC tumors and paired paracancer tissues. Scale: 130 μm. (C) The mRNA levels of B4GALNT3 and B4GALNT4 in ICC tumors (*n* = 33) and normal tissues (*n* = 8) in TCGA database (*P* = 4.5E‐9 for B4GALNT3 and *P* = 1.9E‐5 for B4GALNT4 by Wilcoxon's test). (D) The mRNA levels of B4GALNT3 and B4GALNT4 in HCC tumors (*n* = 364) and normal tissues (*n* = 49) in TCGA database (*P* = 0.13 for B4GALNT3 and *P* = 0.89 for B4GALNT4 by Wilcoxon's test).

To further confirm the increase of LacdiNAc‐containing glycans in ICC, we performed lectin histochemistry analysis on six ICC tumors and their paired paracancer tissues using Cy5‐labeled wisteria floribunda agglutinin (WFA) for the detection of LacdiNAc structures [55, 56]. The histochemical results demonstrated that the binding of WFA was significantly stronger (*P* < 0.01) in ICC tumors compared with their adjacent normal tissues (Fig. 7A). The enhancement of WFA‐reactive glycans in ICC tumors was in agreement with previous reported histochemical results of ICC using biotinylated WFA [17]. The general increase of LacdiNAc structures in ICC tumor was also confirmed by the elevated protein expression of corresponding β1‐4‐acetylgalactosamine transferases B4GALNT3 (*n* = 6, *P* < 0.01) and B4GALNT4 (*n* = 6, *P* < 0.05) [45, 57] measured by the immunohistochemistry assay (Fig. 7B). Consistently, based on the transcriptome data in the TCGA database, the mRNA levels of B4GALNT3 and B4GALNT4 were also significantly elevated (*P* < 0.0001) in ICC tumors (*n* = 33) compared with normal tissues (*n* = 8) (Fig. 7C), while no significant difference was observed in HCC tumors (*n* = 364) compared with the corresponding normal tissues (*n* = 49) (Fig. 7D). Altogether, the above results further confirmed that the LacdiNAc structure was specifically increased in ICC tumors.

## 4 Conclusion

By using our newly developed structural characterization strategy and software strucgp, we comprehensively identified site‐specific N‐glycan structures at glycopeptide level in ICC and HCC tissues. Specifically, site‐specific N‐glycans with uncommon LacdiNAc (GalNAcβ1‐4GlcNAc) structures were distinguished from their isomeric glycans. Integrating with TMT‐based quantification, we uncovered the great differences of site‐specific N‐glycans between tumors (ICC or HCC) and non‐tumors as well as between ICC and HCC tumors. Notably, the majority of LacdiNAc‐containing glycans were specifically increased in ICC tumors, which were further confirmed by lectin histochemistry and the high expression of β1‐4GalNAc transferases in ICC at both protein and mRNA levels. Further validation and functional study, which are still underwent in our laboratory, will allow us to uncover their functional implications in ICC.

Site‐specific glycans have been recognized as important diagnostic biomarkers and therapeutic targets for many diseases including cancers. However, it is highly challenging to precisely characterize glycosylation of proteins. By direct structural characterization of intact N‐glycopeptides, our approach not only identifies functional glycan structures but also identifies the modified glycoproteins. The identified functional glycan structures can be further pursued as targets for specific biomarkers of detection and treatment, and at the meantime, the corresponding glycoproteins allows us to perform further functional investigation. By discovering specific alteration of LacdiNAc structures and modified proteins, we provide a new perspective for distinguishing ICC from HCC and exploring its mechanism. All in all, structural characterization of site‐specific N‐glycans has great potential to identify novel aberrant glycosylation in cancers, which provides the basis on their biomarker discovery and functional investigation.

## Conflict of interest

The authors declare no conflict of interest.

## Author contributions

JL, TZ, and SS designed the experiments; FM and LH collected the tissue samples and clinical information; TZ performed experiments with help from LJ, LD, and BZ; JL analyzed data with the help from JingL, JS, CM, and DL; JL and SS wrote and edited the manuscript; and all the authors contributed to manuscript review.

### Peer review

The peer review history for this article is available at https://publons.com/publon/10.1002/1878‐0261.13147.

## Supporting information


**Fig. S1.** LC chromatograph and MS/MS spectra of glycan isoforms identified using StrucGP.
**Fig. S2.** Enriched cellular component and KEGG pathways of the altered glycoproteins in ICC and HCC tumors.
**Fig. S3.** Commonly altered glycopeptides in both ICC and HCC tumors compared with paracancer tissues with additional differences between ICC and HCC tumors.
**Fig. S4.** Heatmap of the 95 glycopeptides specially altered in ICC or HCC tumors.
**Fig. S5.** Specifically altered glycopeptides in ICC or HCC tumors.
**Fig. S6.** KEGG pathway analyses of specifically altered glycopeptides in ICC tumors.
**Table S1.** The clinical information of ICC and HCC patients involved in this study.
**Table S2.** All identified intact glycopeptides in tumors and paracancerous samples of ICC and HCC.
**Table S3.** All quantified intact glycopeptides in tumors and paracancerous samples of ICC and HCC.
**Table S4.** Altered glycoproteins in ICC or HCC tumors compared with paired paracancerous tissues.
**Table S5.** Commonly altered glycopeptides in ICC and HCC tumors.
**Table S6.** Differentially altered glycopeptides in ICC and HCC tumors.

## Data Availability

The mass spectrometry data have been deposited to the ProteomeXchange Consortium (http://proteomecentral.proteomexchange.org) via the PRIDE partner repository [58] with the dataset identifier PXD020277.
