# Supplementary material for: Precision N‐glycoproteomics reveals elevated LacdiNAc as a novel signature of intrahepatic cholangiocarcinoma
Source: Mol Oncol. 2021 Dec 18;16(11):2135–52. doi: 10.1002/1878-0261.13147 (PMC9168967; doi:10.1002/1878-0261.13147)
Supplement: Supplementary file 1 — Fig. S1. LC chromatograph and MS/MS spectra of glycan isoforms identified using StrucGP. Fig. S2. Enriched cellular component and KEGG pathways of the altered glycoproteins in ICC and HCC tumors. Fig. S3. Commonly altered glycopeptides in both ICC and HCC tumors compared with paracancer tissues with additional differences between ICC and HCC tumors. Fig. S4. Heatmap of the 95 glycopeptides specially altered in ICC or HCC tumors. Fig. S5. Specifically altered glycopeptides in ICC or HCC tumors. Fig. S6. KEGG pathway analyses of specifically altered glycopeptides in ICC tumors. Table S1. The clinical information of ICC and HCC patients involved in this study. Table S2. All identified intact glycopeptides in tumors and paracancerous samples of ICC and HCC. Table S3. All quantified intact glycopeptides in tumors and paracancerous samples of ICC and HCC. Table S4. Altered glycoproteins in ICC or HCC tumors compared with paired paracancerous tissues. Table S5. Commonly altered glycopeptides in ICC and HCC tumors. Table S6. Differentially altered glycopeptides in ICC and HCC tumors. [file MOL2-16-2135-s002.pdf]

Supporting Information for:

Precision *N*-glycoproteomics reveals elevated LacdiNAc as a novel signature of intrahepatic cholangiocarcinoma

Jun Li[1, #], Ting Zhao[1, #], Jing Li[1], Jiechen Shen[1], Li Jia[1], Bojing Zhu[1], Liuyi Dang[1], Chen Ma[1], Didi Liu[1], Fan Mu[2], Liangshuo Hu[2, *], Shisheng Sun[1, *]

[1]College of Life Science, Northwest University, Xi'an, 710069, P. R. China

[2]Department of Hepatobiliary Surgery and Institute of Advanced Surgical Technology and Engineering, The First Affiliated Hospital of Xi'an Jiaotong University, Xi'an, 710061, P. R. China

[#]These authors contributed equally

*__Correspondence:__

Shisheng Sun, Email: suns@nwu.edu.cn;

Liangshuo Hu, Email: huliangshuo1983@hotmail.com;

Table of Contents

1. Supplementary figures

Figure S1. LC chromatograph and MS/MS spectra of glycan isoforms identified using StrucGP.

Figure S2. Enriched cellular component and KEGG pathways of the altered glycoproteins in ICC and HCC tumors.

Figure S3. Commonly altered glycopeptides in both ICC and HCC tumors compared with paracancer tissues with additional differences between ICC and HCC tumors.

Figure S4. Heatmap of the 95 glycopeptides specially altered in ICC or HCC tumors.

Figure S5. Specifically altered glycopeptides in ICC or HCC tumors.

Figure S6. KEGG pathway analyses of specifically altered glycopeptides in ICC tumors.

2. Supplementary tables (as separate files)

Table S1. The clinical information of ICC and HCC patients involved in this study. (.xlsx)

Table S2. All identified intact glycopeptides in tumors and paracancerous samples of ICC and HCC. (.xlsx)

Table S3. All quantified intact glycopeptides in tumors and paracancerous samples of ICC and HCC. (.xlsx)

Table S4. Altered glycoproteins in ICC or HCC tumors compared with paired paracancerous tissues. (.xlsx)

Table S5. Commonly altered glycopeptides in ICC and HCC tumors. (.xlsx)

Table S6. Differentially altered glycopeptides in ICC and HCC tumors. (.xlsx)

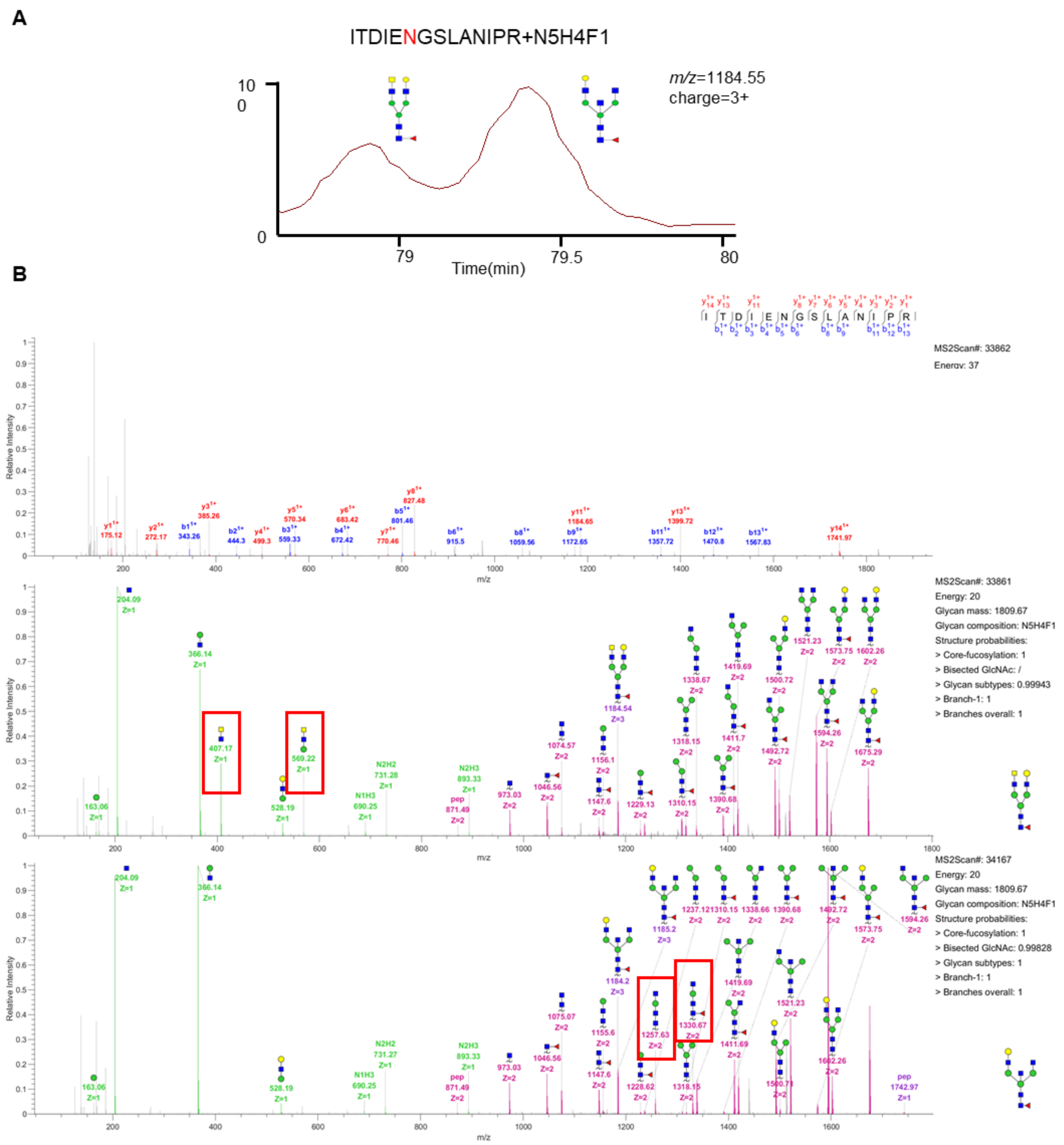

Figure S1. LC chromatograph and MS/MS spectra of glycan isoforms identified using StrucGP. (A) LC separation of MS1 peaks representing different glycan isoforms of the glycan HexNAc5Hex4Fuc1 (N5H4F1) attached at the same peptide (ITDIEN#GSLANIPR). (B) The MS2 spectra of above glycopeptides with two glycan isoforms of N5H4F1. Upper: peptide sequence identification using a MS/MS spectrum with high HCD energy (HCD=37%). Lower: glycan structure identification using MS/MS spectra with low HCD energy (HCD=20%).

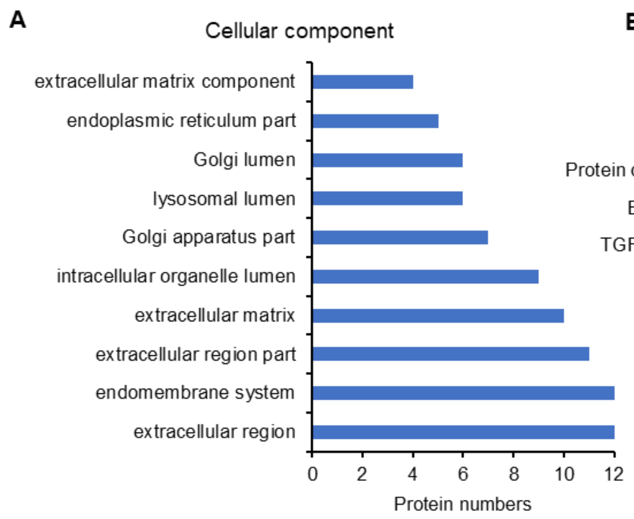

Figure S2. Enriched cellular component (B), and KEGG pathways (C) of the altered glycoproteins in ICC and HCC tumors.

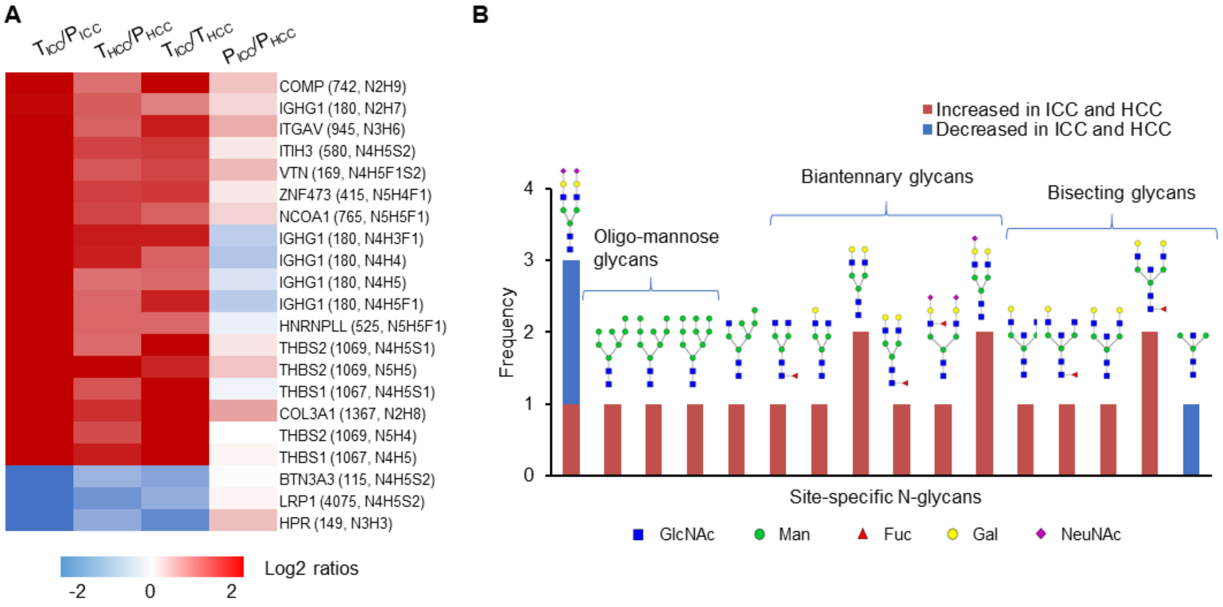

Figure S3. Commonly altered glycopeptides in both ICC and HCC tumors compared with paracancer tissues with additional differences between ICC and HCC tumors. (A) Profiling of 33 increased glycopeptides and 14 decreased glycopeptides in both ICC and HCC tumors. T_{ICC}: tumors of ICC; P_{ICC}: paracancers of ICC; T_{HCC}: tumors of HCC; P_{HCC}: paracancers of HCC. (B) Frequencies of commonly changed site-specific glycans in ICC and HCC tumors.

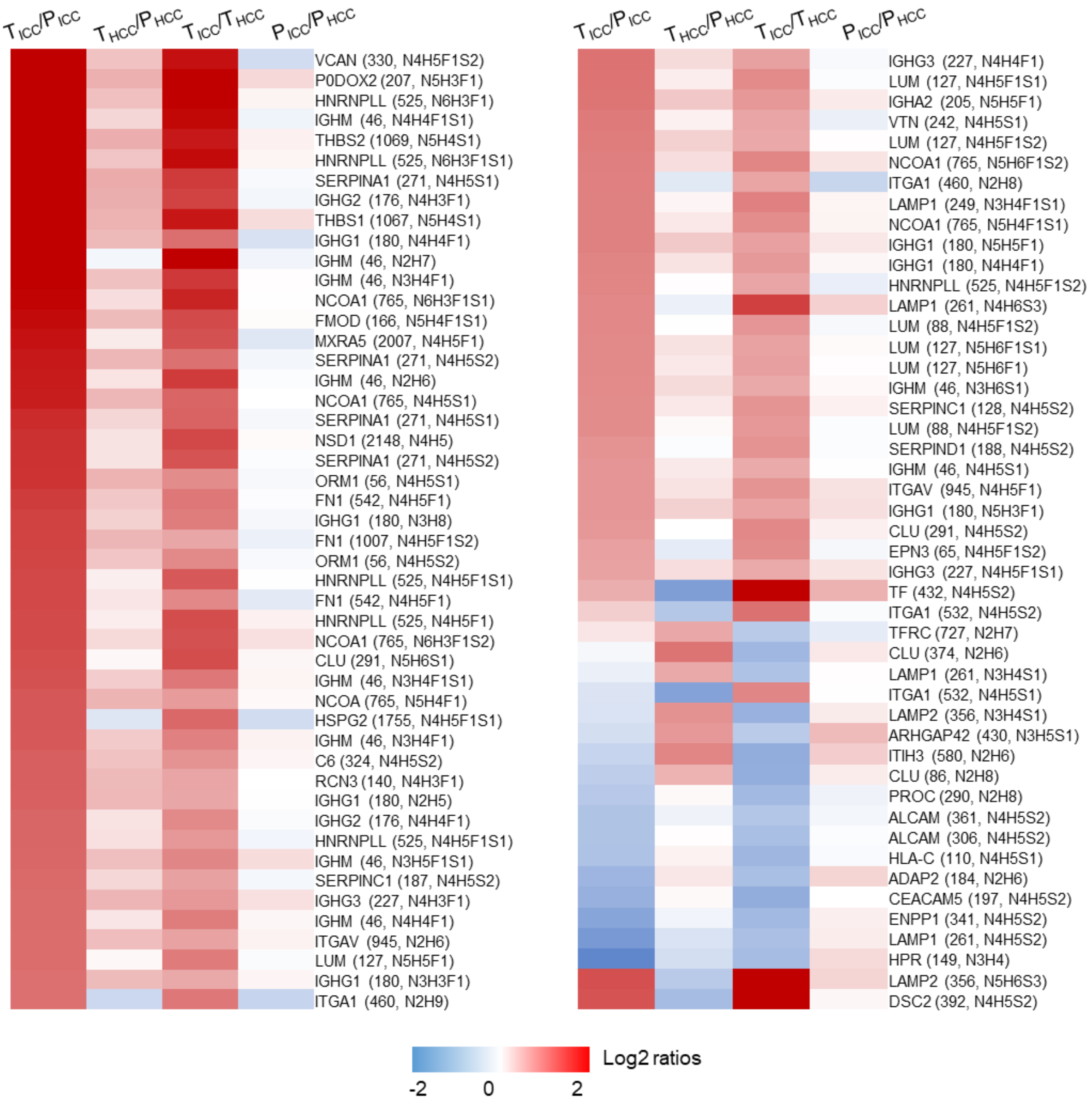

Figure S4. Heatmap of the 95 glycopeptides specially altered in ICC or HCC tumors. The Log2 ratios between groups were calculated based on the normalized abundance of TMT labeling. T_{ICC}: tumors of ICC; P_{ICC}: paracancers of ICC; T_{HCC}: tumors of HCC; P_{HCC}: paracancers of HCC.

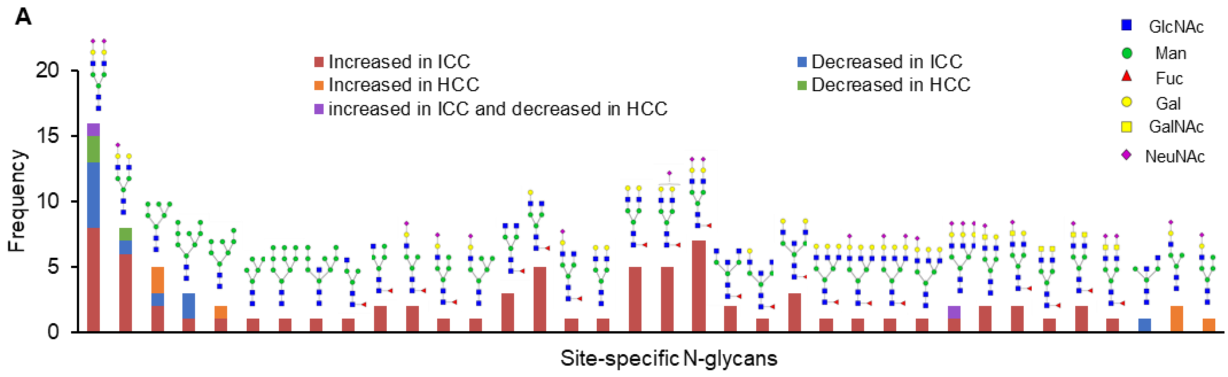

Figure S5. Glycopeptides with altered site-specific glycosylation in either ICC or HCC tumor. (A) Frequencies of site-specific glycans that uniquely altered in ICC and/or HCC tumors: solely increased in ICC (in red), solely decreased in ICC (in blue), solely increased in HCC (in orange), solely decreased in HCC (in green), increased in ICC and decreased in HCC (in purple).

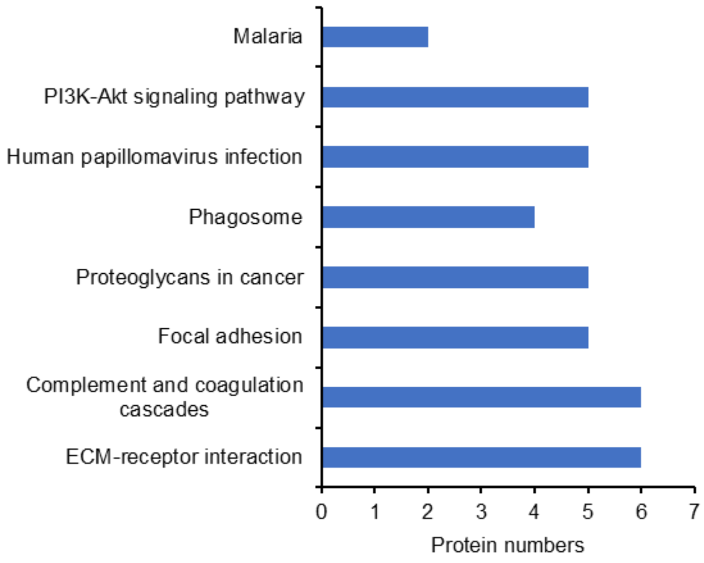

Figure S6. KEGG pathways analysis of the 28 glycoproteins with specific increased site-specific glycosylation in ICC tumors.
